# *In vivo* muscle force and muscle power during near-maximal frog jumps

**DOI:** 10.1371/journal.pone.0173415

**Published:** 2017-03-10

**Authors:** Eng Kuan Moo, Daniel R. Peterson, Timothy R. Leonard, Motoshi Kaya, Walter Herzog

**Affiliations:** Human Performance Laboratory, Faculty of Kinesiology, University of Calgary, Calgary, Alberta, Canada; Universite de Nantes, FRANCE

## Abstract

Frogs’ outstanding jumping ability has been associated with a high power output from the leg extensor muscles. Two main theories have emerged to explain the high power output of the frog leg extensor muscles, either (i) the contractile conditions of all leg extensor muscles are optimized in terms of muscle length and speed of shortening, or (ii) maximal power is achieved through a dynamic catch mechanism that uncouples fibre shortening from the corresponding muscle-tendon unit shortening. As *in vivo* instantaneous power generation in frog hind limb muscles during jumping has never been measured directly, it is hard to distinguish between the two theories. In this study, we determined the instantaneous variable power output of the plantaris longus (PL) of *Lithobates pipiens* (also known as *Rana pipiens*), by directly measuring the *in vivo* force, length change, and speed of muscle and fibre shortening in near maximal jumps. Fifteen near maximal jumps (> 50cm in horizontal distance) were analyzed. High instantaneous peak power in PL (536 ± 47 W/kg) was achieved by optimizing the contractile conditions in terms of the force-length but not the force-velocity relationship, and by a dynamic catch mechanism that decouples fascicle shortening from muscle-tendon unit shortening. We also found that the extra-muscular free tendon likely amplifies the peak power output of the PL by modulating fascicle shortening length and shortening velocity for optimum power output, but not by releasing stored energy through recoiling as the tendon only started recoiling after peak PL power had been achieved.

## Introduction

Frogs have an impressive jumping ability. They are capable of jumping a horizontal distance exceeding ~30 times of their body length [1] by accelerating from a stationary initial position to great speed at takeoff in a fraction of a second. Such an explosive movement requires a high power output from the frog hind limb muscles. However, a conclusive explanation of how frogs achieve their jumping feats remains elusive.

There are two main theories in the literature proposed for explaining the mechanism of the high power generation in frog hind limb muscles during jumping. In the first theory, it is proposed that the hind limb muscles are used under optimal contractile conditions, which include operating at lengths across the plateau region of the force-length relationship and shortening at a constant “optimal” speed that maximizes power production for maximally activated muscles [2]. In this theory all leg extensor muscles must work optimally, and therefore produce an equal power output per volume of muscle. However, the frog has more than fifteen leg extensor muscles [3] that vary in architecture. In particular, the presence/absence of series elasticity (e.g., extra-muscular free tendon, denoted as tendon hereon) may affect the ability for power production [4–8]. The ‘optimal contractile conditions’ theory was derived based on observations on the frog semimembranosus (SM) muscle which has no appreciable tendon. Therefore, this theory may neglect the potential role played by the tendon found in some of the leg extensor muscles.

The second theory takes into account the series elastic element. It is proposed that the series elastic (tendinous) elements can amplify the peak power output of the muscle-tendon unit (MTU) in the takeoff phase of a jump. Using the frog plantaris longus (PL) muscle, fascicle shortening was found to be uncoupled from the shortening of the corresponding MTU during jumping [7,9,10]. This uncoupling event is reminiscent of an anatomical catch mechanism that is found in arthropods (e.g., fleas, locusts and mantis shrimp) which allows the limb muscles to stretch elastic elements prior to movement and store potential energy for later release [11–13]. It has been argued that this dynamic catch mechanism may be used by frogs to amplify peak power production of the MTU by using the stored elastic energy in the late propulsive phase of the jump [7,9,10].

However, the key parameter in these assertions, the muscle mechanical power, has never been measured but was indirectly inferred. Typically, the power generated in the take-off phase of frog jumping has been calculated from the kinetics (ground reaction force, GRF) and kinematics (take-off velocity and jump distance) of the jump [1,2,14–16], or by attempting to recreate the instantaneous contractile conditions of the muscle during jumping in an *in vitro* muscle preparation [2,17]. In none of the in vivo approaches were the muscle forces directly measured, nor has there been any attempt to separate the total frog jumping power among the individual leg extensor muscles [2,18]. As the previously reported muscle forces were estimated based on indirect measurements, there is always uncertainty as to the actual force in a muscle during the jump.

Therefore, the purpose of this study was to determine the power output of one of the major contributors to frog jumping, the PL, by directly measuring its *in vivo* force, length change, and speed of muscle and fibre shortening in near maximal jumps in a laboratory setting. With our direct measurement approach, we can either confirm the findings of previous studies, or find a very different conclusion. In any case, direct force measurements will help us elucidate the mechanism that allows PL to produce the required power for jumping.

## Materials and methods

### Animal preparation

All aspect of animal care and experimental procedures were approved by the University of Calgary committee for the ethical use of animals in research. Ten northern leopard frogs (*Lithobates pipiens*, also known as *Rana pipiens*) were obtained from a commercial supplier (Boreal Science, ON, Canada). Animals were kept in large water-filled plastic containers with several levels of dry platforms. The room was maintained at ~21°C with a 12 h:12 h light-dark cycle, and the water was kept at room temperature. The frogs were fed mealworms *ad libitum*.

**Fig 1:**
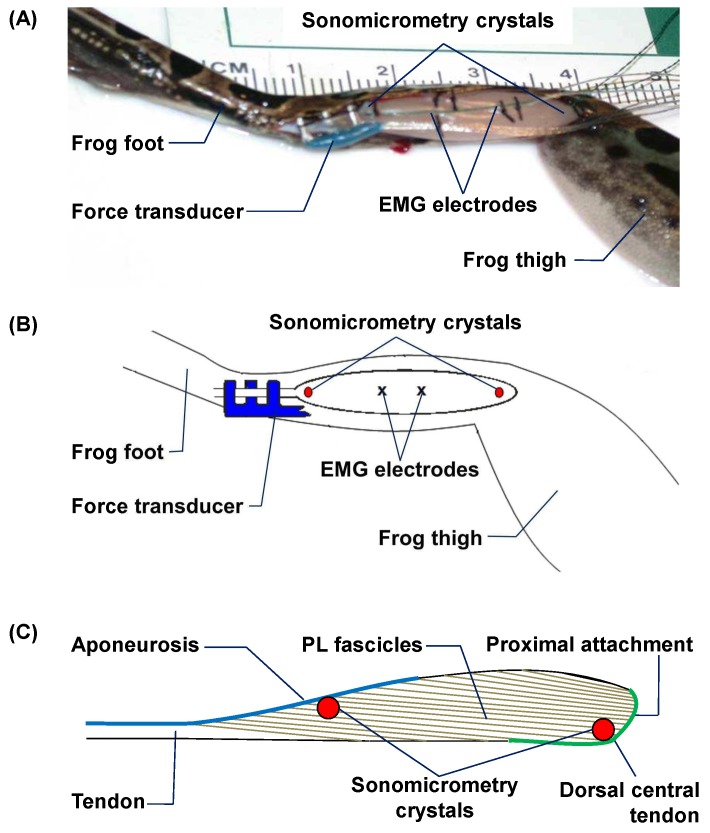
**(A) Digital photograph and (B) schematic illustration of a fully instrumented frog plantaris longus (PL) muscle** prior to closing the incision, which shows an E-shaped tendon force transducer on the PL tendon, a pair of sonomicrometry crystals at the proximal and distal ends of a central fascicle, and two EMG electrodes embedded in the mid-belly of PL. (C) Schematic illustration of the sonomicrometry placement in PL relative to fascicle orientation.

On the day of the experiment, animals were anesthetized by immersion in a 0.5% tricane methylsulfonate (MS-222) solution. The left PL muscle was exposed by making an incision on the posterior side of the lower left shank (Fig 1A). A pair of piezoelectric sonomicrometer crystals (Sonometrics, ON, Canada) of 1mm-diameter was sutured into small pockets of muscle underlying the fascia at the proximal and distal ends (identified by micro-stimulation) of a central fascicle in the PL (Fig 1). Two indwelling, bipolar electromyographic (EMG) electrodes (Cooner Wire, CA, USA) were inserted into the mid-belly of the PL and held in place by suture (Fig 1). A custom-made tendon force transducer (see S1 File) was implanted onto the PL tendon (Fig 1). Following implantation, the bundle of wires from the crystals, EMG electrodes and tendon force transducer were secured to the PL fascia by sutures. The incision was then closed by sutures and the wire bundle was gathered in a loop and sutured to the skin on the frog’s back.

### Experimental procedures

For the experiment, the frogs were administered Buprenorphine at a dosage of 46 μg/ body weight. Frogs were allowed to recover from surgery for 2–3 hours in an aquarium. Then, frogs were placed on a custom-built GRF platform (7x7cm^2^) for measurement of vertical and anterior-posterior GRFs. All testing took place at room temperature. Animals were videotaped using a lateral view, high-speed video camera (MotionScope, Redlake Imaging, CA, USA) operating at 1000 frames/s. The resting lengths of the MTU and fascicle were determined when the frog was sitting still on the GRF platform prior to jumping. Then, the frog was encouraged to jump by lunging toward the frog with a hand extended and lightly blowing in the direction of the frog.

During frog jumping, the signals from sonomicrometry, EMG, tendon force transducer, GRF platform and digital video were collected simultaneously and synchronized with square wave pulses delivered simultaneously to the recording computers. Sonometric data were collected at 300 Hz. EMG signals, PL muscle forces and GRFs were collected at 500 Hz, respectively, through Windaq software (Dataq Instruments, OH, USA). The EMG signals were band-pass filtered with cut-off frequencies of 15 and 500 Hz.

Following the experiment, frogs were sacrificed by double pithing. Calibration of the tendon force transducer (see S2 File) was performed as described previously [19,20].

### Jumping kinematics and kinetics

The take-off phase of each jump was defined as the duration starting from the instant when the resultant GRF exceeded body weight until the instant when the resultant GRF became zero. The frog jumping event was divided into equal segments of 10ms. The hip, knee, ankle, tarsometatarsal, and metatarsophalangeal joints were manually digitized and analyzed for joint angle changes during jumps using a custom-designed MATLAB program (Math Works Inc., MA, USA). Rotation of the legs from the sagittal plane was measured with a top-view camera and PL length and shortening velocity were corrected for these out of plane movements [21]. The take-off angle was determined as the angle from the horizontal axis of the line connecting the centre of mass of the frog when it first entered the aerial phase to that of when the frog was at its initial position.

### Muscle length change, velocity, power and EMG analysis

A 'tendon travel' approach (see S2 File) was used to measure the PL MTU length as a function of ankle and knee joint angles [22]. Length changes of the PL MTU during jumping were then determined by combining the results of joint angle changes obtained from the kinematic analysis of the jumps and the empirical relationship of MTU lengths as a function of joint angles. The resulting data of MTU length changes with respect to time were best-fitted to a quintic-spline curve. The MTU shortening velocity, $\vec{v_{\_PL}}$, was then determined by differentiating this best-fitting quintic-spline curve with respect to time.

The normalized mechanical power of the PL muscle–tendon unit, *P*_*_MTU*_, was determined by:

$$P_{\_MTU}=(\vec{F_{\_PL}}\times \vec{v_{\_PL}})/m_{\_PL}$$ (1)

where

*m*__*PL*_ is the mass of the plantaris longus
$\vec{F_{\_PL}}$ is the instantaneous PL force measured from the tendon force transducer
$\vec{v_{\_PL}}$ is the corresponding instantaneous shortening speed of PL MTU

Also, the normalized mechanical power of the frog, *P*__*body*_, for the jump was determined from the GRFs based on the following formulas [16]:

$$\vec{a_{\_body}}=(\vec{GRF}-\vec{body\_weight})/m_{\_body}$$ (2)

$$P_{\_body}=(\vec{GRF}-\vec{body\_weight})\times \vec{v_{\_body}}/m_{\_body}$$ (3)

where

*m*_*_body*_ is the mass of the frog
$\vec{GRF}$ is the resultant ground reaction force
$\vec{a_{\_body}}$ is the instantaneous acceleration of the frog’s centre of mass
$\vec{v_{\_body}}$ is the instantaneous velocity of the frog’s centre of mass derived by the integration of the $\vec{a_{\_body}}$

No in-depth analysis was performed on the EMG signals as they were only used to determine the onset and offset times of the muscle activity during jumping. Muscle onset and offset timings were determined by setting a threshold value that was equal to the baseline noise plus two times the standard deviation (SD) of the EMG signal noise at rest. Once this threshold was exceeded (onset) or the EMG fell below that threshold (offset), the muscle was set to be active and the time of activation was determined between these two values.

### Sarcomere length measurement

The left hind limbs were chemically fixed at knee and ankle angles corresponding to the initial position of the jump. After the PL muscles were digested in 30% nitric acid, six fibres were dissected from the centre region of the muscles. Fibre lengths were determined using video analysis software (Matrox Systems Inc., QC, Canada). Average sarcomere length was determined for five regions of a fibre (N_fiber_ = 6) using a laser diffraction technique. Briefly, isolated individual fascicles were placed under a monochromatic Helium-Neon laser (JDS Uniphase Corp., Milpitas, USA) and illuminated by a 5mW laser beam of 0.8mm-diameter at 633nm wavelength to generate diffraction patterns. Average sarcomere lengths were evaluated by measuring the distance between the zeroth- and first- order diffraction [23]. The number of in-series sarcomeres (S_n_) was obtained by dividing the average fibre length by the average sarcomere length. The range of average sarcomere lengths during jumping was calculated by dividing the instantaneous fibre lengths (measured by sonomicrometry during jumping) by S_n_ [24].

## Results

As we were interested in how frogs achieve their extraordinary jumps, only near maximal jumps were included for analysis in this study (see S3 File for all raw data). Data were collected from two frogs (average snout-vent length of 6.4cm) and 15 jumps in total. All data were expressed as means ± standard error of the mean (SEM). In the current analysis, 12 near maximal jumps (horizontal jumping distance > 50cm) obtained from one frog and 3 near maximal jumps from another frog were considered. The average jump distance was 59±1cm (> 9 times the frogs’ body length) at a take-off angle of 54±1° from the horizontal axis. Table 1 summarizes relevant data regarding the muscle properties and jumping performance of the frogs, MTUs and muscle fascicles.

**Table 1:** Muscle properties of plantaris longus (PL) and jumping performance of frogs. Only near maximal jumps (jumps of > 50cm) were included in the analysis. The muscle properties were averaged across all animals tested (N_muscle_ = 2), while the jumping performances were averaged across all analyzed jumps (N_jump_ = 15). Data were expressed in mean ± standard error of the mean.

	Whole frog			Muscle-tendon unit of PL			Fascicle of PL		
**Muscle properties**									
Mass (g)	26.0	±	0.2	0.5	±	0.0		—	
Length (mm)		—		32	±	1	17.7	±	0.1
PCSA(mm^2^)		—		28.6	±	0.5		—	
Angle of pennation (°)		—			—		8.2	±	0.5
**Jumping Performance**									
Jump distance (cm)	59	±	1		—			—	
Take-off angle (°)	54	±	1		—			—	
Duration of activation (ms)		—		97	±	11		—	
Duration of propulsive phase (ms)	109	±	4		—			—	
Peak vertical ground reaction force (N)	1.00	±	0.04						
Time to peak force (% of prop. phase)		—		70	±	1		—	
Shortening length (mm)		—		3.7	±	0.2	1.4	±	0.1
Peak muscle force (N)		—		3.3	±	0.2		—	
Peak muscle stress (kNm^-2^)		—		114	±	5		—	
Peak shortening velocity (mm/s)		—		88	±	4	25	±	1
Normalized peak power of frog, *P*_*_body*_ (W/kg)	73	±	3		—			—	
Normalized peak power of MTU, *P*_*_MTU*_ (W/kg)		—		536	±	47		—	
Range of normalized power (W/kg)	54	—	88	256	—	753		—	

**Fig 2:**
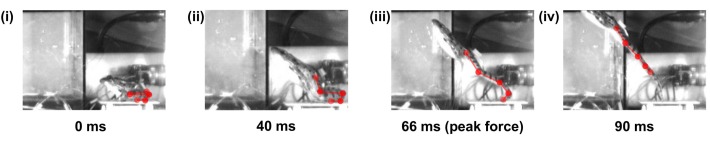
Sequential event of a 65cm jump by a 26g frog. The images represented lateral views and were displayed chronologically from left to right: (i) beginning of the propulsive phase (the time when resultant GRF exceeds 1 body weight), (ii) the moment when the PL MTU started to shorten, (iii) the moment when maximal force was recorded by the tendon force transducer, (iv) end of the propulsive phase (the time when the frog completely left the force plate and started the aerial phase). The lower limb joints (hip, knee, ankle, tarsometatarsal, and metatarsophalangeal) were digitized and connected with superimposed red lines to show the changes in hind limb geometry throughout the take-off phase.

**Fig 3:**
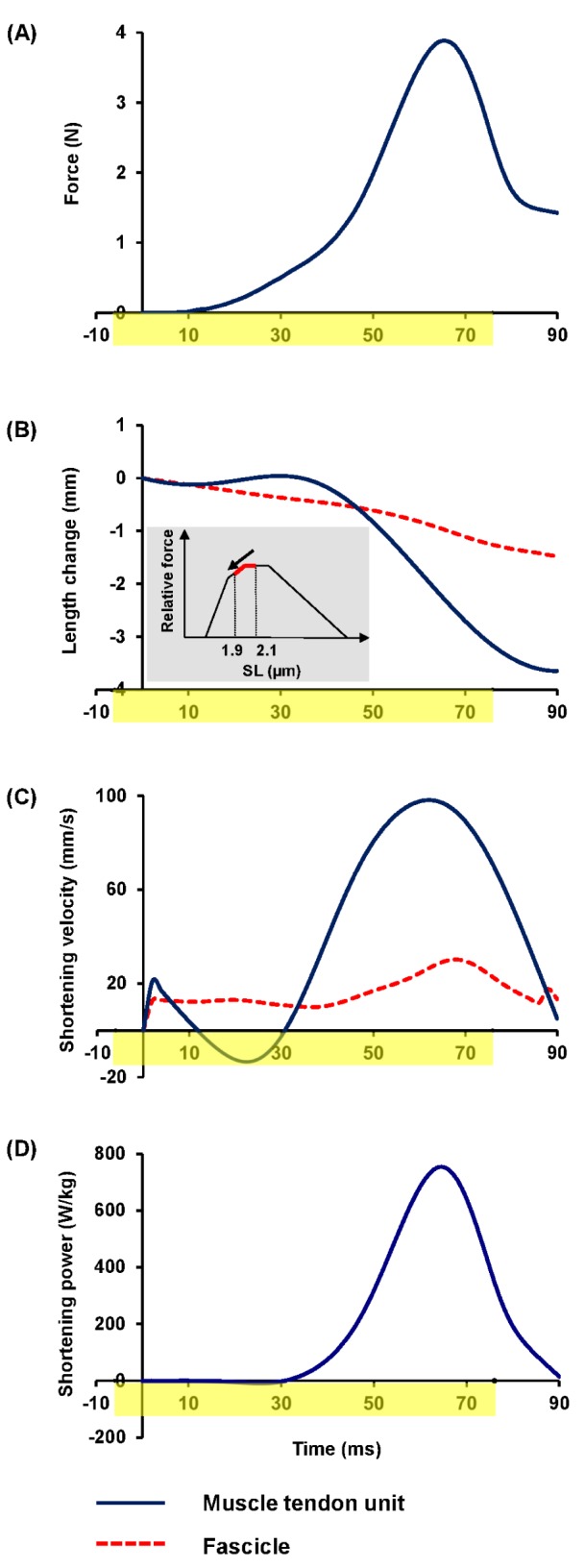
**Representative curves of (A) muscle force, (B) length changes, (C) shortening velocity, and (D) shortening power of a plantaris longus (PL) muscle (black solid curves) and/or fascicle (red dashed curves) during a 65cm jump as shown in Fig 2**. The duration of muscle activation is indicated by the yellow-highlighted region on the time axes. Time zero is defined as the beginning of the propulsive phase (see ‘Methods’ section for details). The inset in (B) shows the sarcomere force-length curve of a frog [41], with the sarcomere range observed in the current study highlighted by a bold red line (from 1.91±0.08 μm to 2.07±0.09 μm; N_fiber_ = 6). Peak shortening power occurred when peak muscle force and peak shortening velocity were achieved.

Fig 2 depicts sequentially the important events occurring during the propulsive phase of a representative jump. Overall, the muscle underwent a concentric contraction during the takeoff. The fascicle started shortening from the start (0ms), while the PL MTU stayed at an almost constant length. At 40ms, MTU length started to shorten. At 66ms, peak force was observed in the PL muscle. Peak GRF during the propulsive phase was 1.00±0.04N (~4 times of body weight). Peak PL force during near maximal jumps was 3.3±0.2N (~13 times of body weight) and the corresponding peak muscle stress was 114±5kNm^-2^ (Table 1). The propulsive phase lasted for 109±4ms and peak muscle force was achieved at 70±1% of the propulsive phase. Fig 3A shows a representative time history of PL force recorded from a 65cm jump with the EMG activation phase included.

During jumping, fascicle excursion is smaller than MTU excursion (Fig 3B). Fascicles shortened by 1.4±0.1mm, or ~8% of the resting length while the PL MTU shortened by 3.7±0.2mm (Table 1). The inset in Fig 3B shows the sarcomere force-length relationship for frog skeletal muscle, with the red region indicating the range of sarcomere lengths encountered during frog jumping in the current study (from 2.07±0.09 to 1.91±0.08μm; N_fiber_ = 6).

The peak shortening velocity of the fascicles was 25±1mm/s, or about 1.4 fascicle lengths (FL)/s (Table 1). The peak shortening velocity of the PL MTU was 88±4mm/s, or about 2.8 MTU lengths (ML)/s (Table 1). An example of fascicle and MTU shortening speed for the 65cm jump is shown in Fig 3C.

The body-mass-specific power for the whole frog was 73±3W/kg (Table 1). The muscle-mass-specific power for the PL MTU was 536±47W/kg (Table 1). A representative PL power-time curve for the 65cm jump is shown in Fig 3D. Peak shortening power occurred when peak muscle force and peak shortening velocity were achieved.

## Discussion

**Fig 4:**
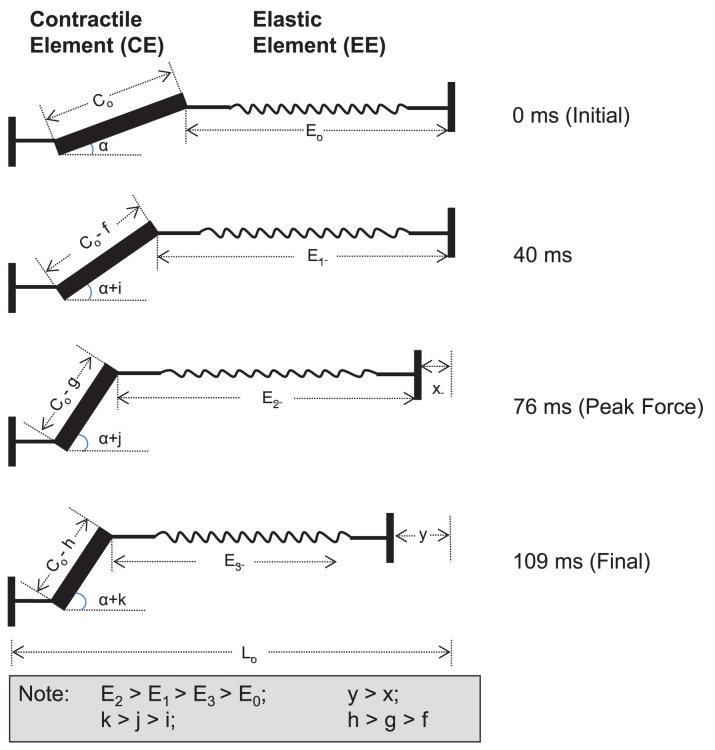
Schematic illustration depicting the proposed length changes of the contractile element (CE, representing muscle fascicles) and elastic element (EE, representing tendon) of plantaris longus (PL) muscle during the propulsive phase of a frog jump made based on the observations in the current study. The length of the PL muscle-tendon unit, L_o_, remains constant in the early part of the propulsive phase, and only starts to decrease after 40ms when it continues to shorten until the end of the propulsive phase. However, the contractile element length, C_o_, shortens throughout the propulsive phase. Likewise, the angle of pennation, α, increases throughout the entire propulsion phase [31]. Due to the series arrangement and the passive property, the series elastic element is stretched continuously in the propulsive phase until the peak muscle force is reached at 76ms. This is followed by a shortening of the EE. At the end of the propulsive phase (109ms), the EE length is shorter than E_2_ and E_1_, but remains longer than the initial EE length, E_o_. Note that the timings shown in the figure are mean values presented in Table 1.

In the current study, near maximal frog jumping (>9 times the frogs’ body length) was investigated through direct measurement of PL muscle forces using a novel force buckle transducer adapted from an earlier design and optimized for use in frogs [19,25]. Our results suggest that the high peak power output by PL is produced through a dynamic catch mechanism during the propulsive phase of jumping [9]. In agreement with previous studies [7,9,10], we found that the shortening of fascicles was uncoupled from the shortening of the MTU (Fig 3B). This is illustrated schematically in Fig 4. While the fascicle shortened throughout the propulsive phase, the MTU’s length remained constant for about the first 40ms of the propulsive phase with a corresponding increase in PL muscle force. After this initial ‘catch’, the MTU started to shorten at a faster speed compared to the fascicle shortening speed. However, the PL muscle force continued to increase and the elastic elements in series, therefore, must be increasing in lengths as well until ~70% of the propulsive phase (Table 1) when peak muscle force was achieved. This is followed by the release of stored elastic energy through a recoiling of the series elastic elements when muscle force decreases.

**Fig 5:**
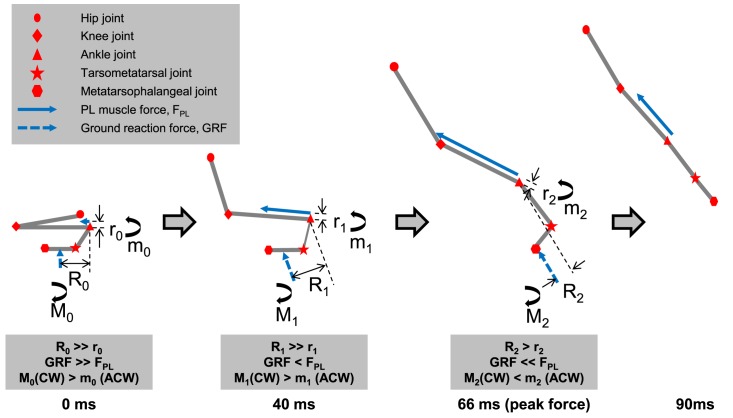
Sequential free body diagrams of frog jumping drawn according to Fig 2 to illustrate the interplay between the plantaris longus (PL) extensor moment, and the ground reaction force (GRF) flexor moment at the ankle joint during jumping. R_i_ and r_i_ (i = 0, 1, 2) are the ground reaction force moment arms and PL muscle moment arms relative to the ankle joint, respectively, and are estimated based on previous studies [7, 9, 10]. M_j_ and m_j_ (j = 0, 1, 2) are the ground reaction force flexor moments and PL muscle extensor moments about the ankle joint, respectively. The effective mechanical advantage (EMA) is defined as the ratio of r_i_ to R_i_. In the first 40ms of the propulsive phase, the GRF flexor moment is bigger than the PL extensor moment. Therefore, virtually no movement is produced due to the joint constraints although the frog’s joints are slightly flexed almost like a small counter movement. The ankle joint angle and PL muscle-tendon unit length remain almost constant in the first 40ms, although there is a slight stretch of the PL MTU from about 10-35ms in Fig 3B, which attests to the slight compression of the frog. After 40ms, the PL extensor moment exceeds the GRF flexor moment, thus causing ankle extension and shortening of the PL muscle-tendon unit. This process is accompanied by an increase in EMA [7,9], with constant r_i_ [10] but a decrease in R_i_. CW–clockwise; ACW–anticlockwise.

During the dynamic catch phase, the ankle joint needs to be restrained from extending in the early part of the propulsive phase to allow for loading of muscle elastic elements. Qualitative analysis of the GRF flexor moments and the PL extensor moments at the ankle provides a possible explanation (Fig 5). For the first 40ms of the propulsive phase, the ankle extensor moment of the PL was smaller than the flexor moment produced by the GRF on the foot. Therefore, the PL fascicles shortened, while the ankle joint angle, and consequently the PL MTU length, remained about constant. At about 40ms into the propulsive phase, the ankle extensor moment produced by the PL started to exceed the flexor moment of the GRF. The ankle began to extend, and the PL MTU started to shorten.

The extra-muscular tendon (i.e., series elastic element) has been thought to play an essential role in amplifying peak power output of the MTU [9,10,26]. Specifically, the tendon is thought to store energy during the initial ‘catch’ phase, and release the stored elastic energy by recoiling in a short duration to enhance the peak power of the MTU. For this mechanism to work, the tendon has to start recoiling before the MTU’s peak power is achieved. We found that the peak force and the peak shortening speed of the muscle always occurred at about the same instant in time (Fig 3). Therefore, by definition, peak power also occurred at about that instant in time (i.e. about 70% into the propulsive phase, Fig 3). Since elastic elements in series with the contractile muscle must increase in length, and therefore cannot produce positive mechanical power as long as muscle force increases, release of potential energy in series elastic elements probably did not contribute to the peak power output, but might have contributed to increased power output in the latter part of the jump once peak PL forces are achieved.

However, PL muscle alone can only produce peak power ranging between 200–300 W/kg [2,26], which is >2 times lower than the peak power output of the PL MTU (536 W/kg, Table 1). Previous in vitro [27] and in vivo [28] studies found that a pre-stretch of the series elastic tendon prior to active shortening of a MTU increases the stiffness of the MTU and, therefore, increases the rate of force production and peak force production by the MTU. In the case of frog jumping, the initial catch in the propulsive phase resulted in longitudinal stretching of the tendon, and biaxial deformation of the aponeuroses [29] prior to MTU shortening. This allows for modulation of the fascicle shortening length and shortening speed for optimal power output. Indeed, the fascicle shortening speed was lower and much more constant than the PL MTU shortening speed (Fig 3B and 3C). In view of the classic force-velocity relationship [30], a slower fascicle shortening speed implies that higher muscle force can be achieved. Therefore, the fascicle shortening speed is arguably more important than the shortening speed of the MTU, as the latter incorporates deformation associated with elastic elements in series (free tendon) and not in series (aponeuroses) with the contractile elements (fascicles).

In several studies, the peak power of frog muscles during jumping has been estimated to range from 371 W/kg for semimembranosus of *Rana pipiens* [2] to 1644 W/kg for all extensors of *Osteopilus septentrionalis* [15]. These previously reported peak power values varied greatly, presumably due to the differences in animal species muscle types, and maybe most importantly, the lack of direct muscle force measurements. In previous studies, total frog power measurements were used to estimate muscle power by distributing the total power evenly over all the extensor muscles [1,2,15]. These methods require assumptions as to which muscles are involved in jumping and to what extent a muscle contributes to the total power output. In the current study, we examined these assumptions by direct measurement of muscle force, fascicle length and MTU length. If we assume that all hind limb extensor muscles (4.4 g, Table 1) produce the same muscle-mass-specific peak power as PL (536 W/kg, Table 1) at the same instant in time, the total peak power normalized to the frog’s body mass (26 g, Table 1) would be 91 W/kg, which is higher than the mean peak power value measured for the 15 near maximal jumps analyzed in this investigation (73 W/kg, Table 1). Likely, not all hind limb muscles need to produce as much power as PL to achieve the observed jumping performance, or alternatively peak power in the various jumping muscles does not need to occur at the same instant in time.

We found that the PL MTU shortens at variable speeds throughout the propulsive phase and at a faster speed than the speed of shortening of the PL fascicles after the initial 'catch' (Fig 3C). This could be due to an increase in the angle of pennation [31–34], shortening of the aponeurosis prior to peak force occurrence [35,36], and/or shortening of in series elastic elements after peak force occurrence. In all cases, however, the muscle shortening speeds (~2.8 ML/s at 21°C, Fig 3) that produced peak muscle power output was slower when compared to the optimal shortening speed found when using the classical force-velocity relationship (3.4 ML/s at 25°C) [2].

Not only was the PL MTU speed of shortening not constant during the propulsive phase of jumping in our frogs, neither was the force. The force rises from zero at the onset of the jump to a maximum at about 70% into the propulsive phase, and then declines. This force-time profile has also been observed for leg extensor muscles of swimming frogs [37] and other animals during jumping and hopping, for example, in the cat soleus and medial gastrocnemius [20,38] or in the medial gastrocnemius muscle of the wallaby [39]. These patterns of muscle force production recorded *in vivo* during jumping differ vastly from those obtained for simulated in vitro conditions, in which forces were found to be constant [2,29] or monotonically decreasing [26]. Thus, these in-vitro results should be considered with caution, as they likely do not represent what happens in vivo.

We also found that the MTU peak shortening velocity was ~4 times faster than that of the fascicle. There are at least two factors that contribute to muscle shortening aside from the shortening of the fascicles. These are the increase in the angle of pennation of fascicles with increasing force [31] and the shortening of elements that are structurally but not mechanically in series with the fascicles, such as the aponeuroses [35,36]. It is well established that fascicle shortening only accounts for ~50% of MTU shortening in skeletal muscles [26,29,40], especially for skeletal muscles with high series compliance. In the current study, we found that the fascicle shortening length was merely ~38% of the MTU shortening length. The detailed reasons for this remain to be explored, but the results are consistent with previous studies in which MTU and fascicle shortening have been measured simultaneously [26,29,40]. Similar shortening velocity profiles of fascicles and MTU's have been observed in human vastus lateralis [40], bullfrog plantaris [26] and lateral gastrocnemius of a turkey [29]. As the peak MTU shortening velocity occurs at about the same instant of the peak MTU force, the tendon in series cannot recoil during this period and therefore cannot contribute to the MTU shortening velocity. However, the aponeurosis and angle of pennation can help in shortening of the MTU in this phase of jumping. Finally, due to the initial 'catch', fascicles could shorten over a longer period of time than MTU (Fig 3). As a result, fascicles shorten at ~25% of the MTU shortening speed.

There are limitations in this study that need to be considered when interpreting our results. First, the sample size used in the current study is small (N = 2 frogs). Although ten frogs were studied in total, not all of them achieved jumping performances that satisfied our *a priori* inclusion criteria, which was to perform jumps of >50cm (near maximal jumps). Second, since 12 out of the 15 analyzed jumps came from one frog, the results presented here may be biased towards one frog. However, the results of the second frog that produced these extreme jumps agreed well with those obtained from the first frog, and there is no inherent reason to believe that frogs use different strategies when performing near maximal jumps. Finally, in order to fully understand the increased shortening speed of the PL MTU compared to the fascicles while PL force is increasing, and thus series elastic elements are still being stretched and do not contribute to the MTU shortening speed, measurements of the angle of pennation and the length of elastic elements not in series with the contractile elements (aponeuroses) are needed. Unfortunately, neither we nor others have ever made such measurements in frog jumping.

In summary, power output of a frog jumping muscle was measured for the first time *in vivo*. The high power output is achieved by a dynamic catch mechanism that involves a decoupling of fascicle and MTU shortening [9]. The high peak power output of PL is associated with optimizing the contractile conditions in terms of the force-length relationship as sarcomeres operate around the plateau region (Fig 3B), but not in terms of the force-velocity relationship as the speed of MTU shortening is not constant and is slower than the optimal PL shortening speed [2]. Although the fascicle shortening speed was virtually constant in the take-off phase, it was also too low for maximal power production [26]. According to our measurements, the series elastic element is likely not involved in peak power amplification of the MTU. Finally, previous assumptions of equal power distribution over all leg extensor muscles used to estimate muscle mass-specific power output should be used with caution as it would result in an underestimation of the actual contribution of the PL.

## Supporting information

S1 File E-shaped tendon force transducer–design and calibration. Details of the design of the tendon force transducer and the steps involved for the calibration of the transducer.
(DOCX)

S2 File Tendon travel approach. Detail steps involved in ‘tendon travel’ approach that is used to derive the empirical relationship of the length of the muscle-tendon unit and the ankle joint angle.
(DOCX)

S3 File Raw data set. Full raw data set of the current study.
(ZIP)
