# Supplementary material for: In vivo muscle force and muscle power during near-maximal frog jumps
Source: PLoS One. 2017 Mar 10;12(3):e0173415. doi: 10.1371/journal.pone.0173415 (PMC5345813; doi:10.1371/journal.pone.0173415)
Supplement: S1 File — Details of the design of the tendon force transducer and the steps involved for the calibration of the transducer. (DOCX) [file pone.0173415.s001.docx]

**Supplementary Materials**

*S1. E-shaped tendon force transducer – design and calibration*

A custom-made tendon force transducer is built based on the design of Herzog *et al*. (1993) and the conceptual idea of Walmsley *et al.* (1978). The tendon force transducer was made of an E-shaped stainless steel (316 alloy) base element instrumented with two strain gauges (model ESU-60-350, Entran Devices Inc., NJ, USA) in a half Wheatstone bridge configuration. The plantaris longus (PL) tendon was woven through the three arms of the transducer and was secured to the transducer by closing the open sides of the E-shaped element with a suture (Fig. 1).

In order to calibrate the tendon force transducer, the PL tendon attached to the tarsal bone was dissected at the distal end and known weights were then hung from the free end of the tendon. The resulting voltage signals from the force transducer showed strong linear correlation with the applied force (r > 0.97).
