# Supplementary material for: In vivo muscle force and muscle power during near-maximal frog jumps
Source: PLoS One. 2017 Mar 10;12(3):e0173415. doi: 10.1371/journal.pone.0173415 (PMC5345813; doi:10.1371/journal.pone.0173415)
Supplement: S2 File — Detail steps involved in ‘tendon travel’ approach that is used to derive the empirical relationship of the length of the muscle-tendon unit and the ankle joint angle. (DOCX) [file pone.0173415.s002.docx]

**Supplementary Materials**

*S2. Tendon travel approach*

The left hind limb of the frog was placed in ankle and knee configurations corresponding to the final position of the jump with PL at its shortest length. At this limb position, the distal tendon of the plantaris longus (PL) was detached from the bone and cut at the insertion point. Using the results of the kinematic analysis described in the main text, the leg was carefully moved through knee and ankle joint configurations that correspond to the joint positions of every 10ms of the jump. The corresponding MTU length changes were measured from the gap distance travelled by the PL tendon with the force always zero (Fig S1).


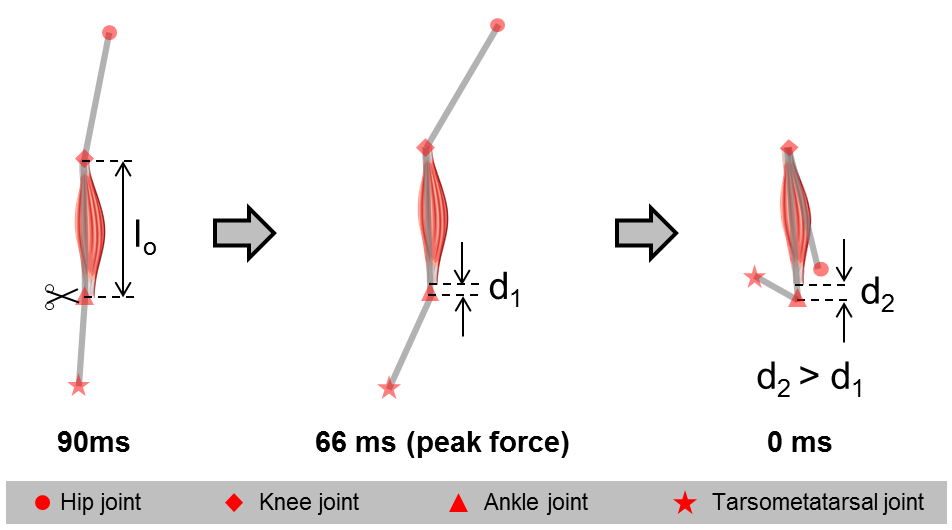


Fig S1: Schematic illustration depicting the ‘tendon travel’ approach used to measure the PL MTU length as a function of ankle and knee joint angles. The free body diagrams are re-drawn based on Fig 5 in the main text, but rotated such that the PL is vertical. The protocol starts with the left hind limb of the frog placed in ankle and knee configurations corresponding to the final position of the jump (90ms) as PL was at it shortest length, l_o_. At this limb position, the distal tendon of PL was severed at the insertion point. Using the video kinematics of ankle and knee, the leg was moved through knee and ankle joint configurations that correspond to the joint positions of every 10ms of the jump. The corresponding MTU length changes were measured from the gap distance travelled by the PL tendon with the force always zero. For instance, MTU length at the start of the jump (0ms) was l_o_ + d_2_.
