# Supplementary figures and images for: In vivo muscle force and muscle power during near-maximal frog jumps
Source: PLoS One. 2017 Mar 10;12(3):e0173415. doi: 10.1371/journal.pone.0173415 (PMC5345813; doi:10.1371/journal.pone.0173415)

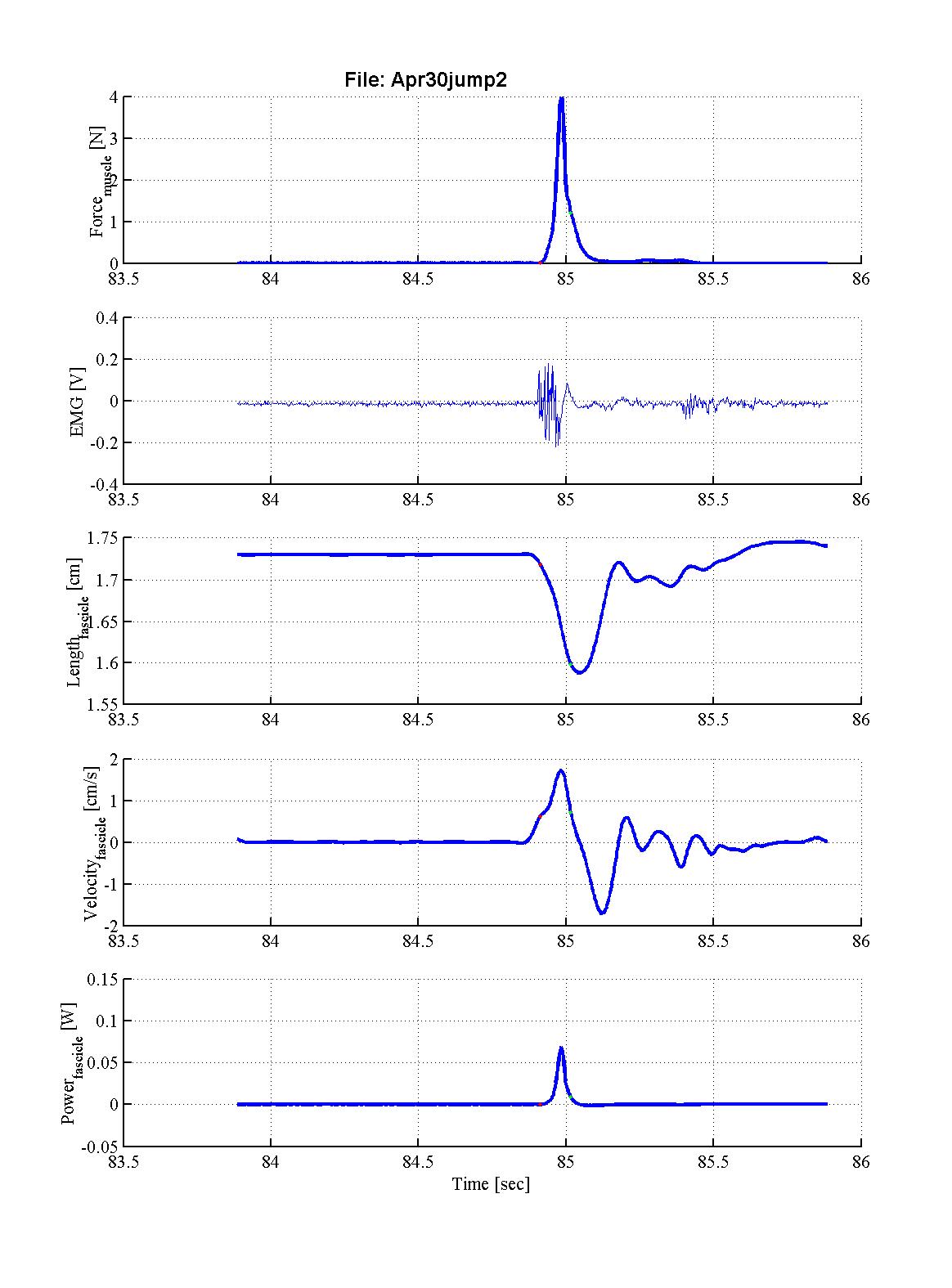

Supplement: S3 File — Full raw data set of the current study. (ZIP) [file pone.0173415.s003.zip › Frog_jumping_full_dataset2/Extracted_data/Frog2/Jump2/allfive.jpg]

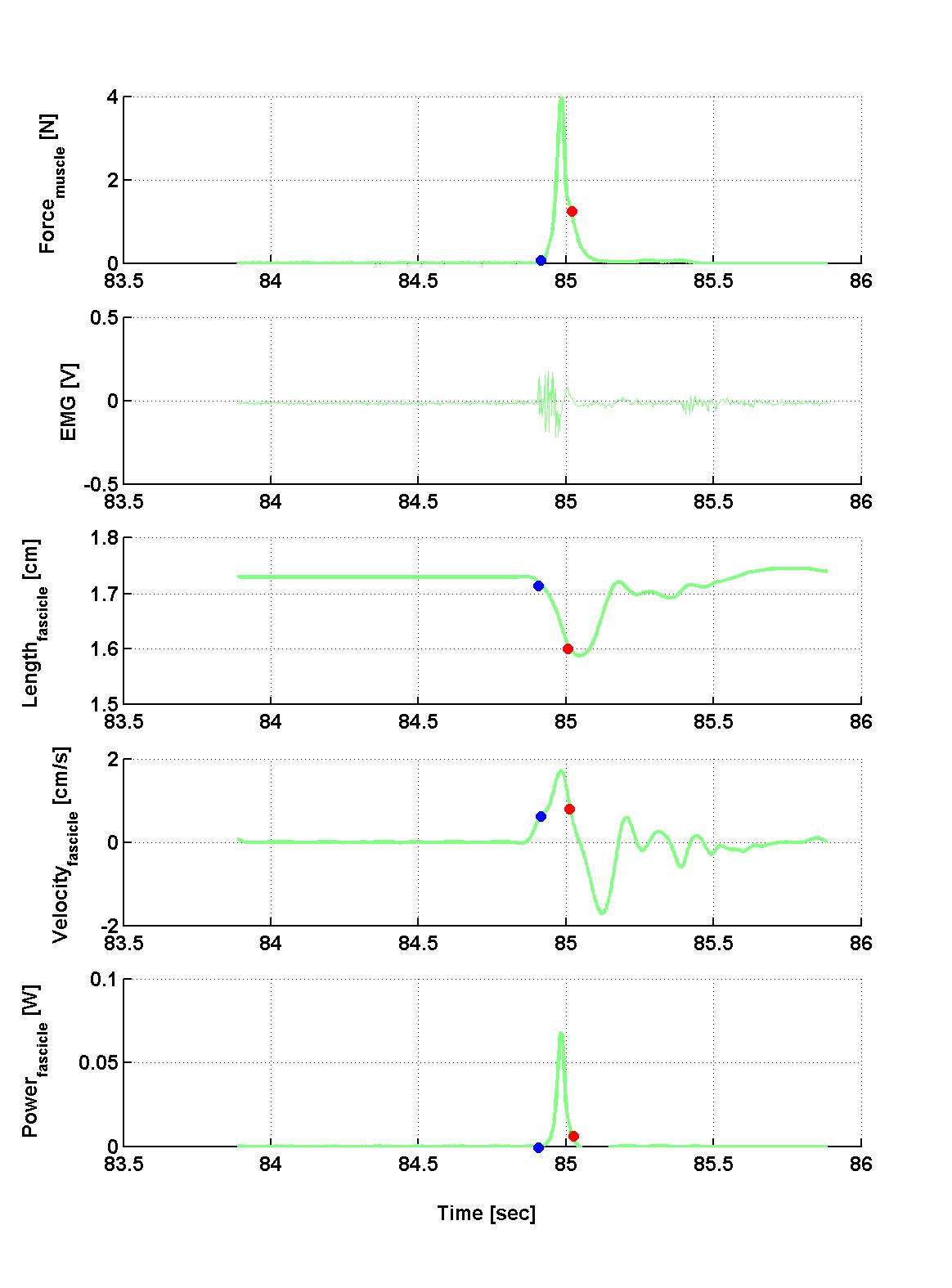

Supplement: S3 File — Full raw data set of the current study. (ZIP) [file pone.0173415.s003.zip › Frog_jumping_full_dataset2/Extracted_data/Frog2/Jump2/blackallfive.jpg]

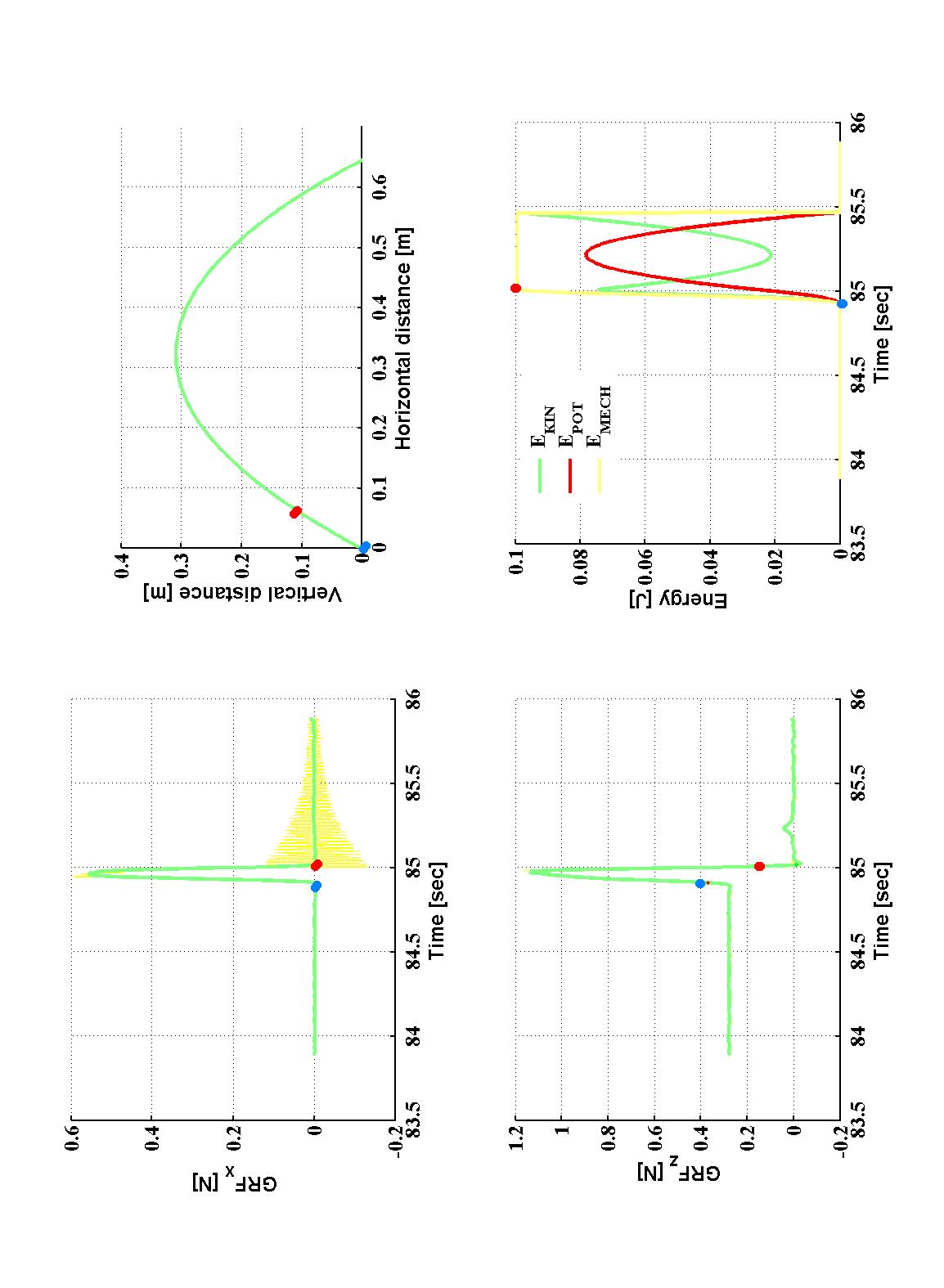

Supplement: S3 File — Full raw data set of the current study. (ZIP) [file pone.0173415.s003.zip › Frog_jumping_full_dataset2/Extracted_data/Frog2/Jump2/blackfp-dis-en.jpg]

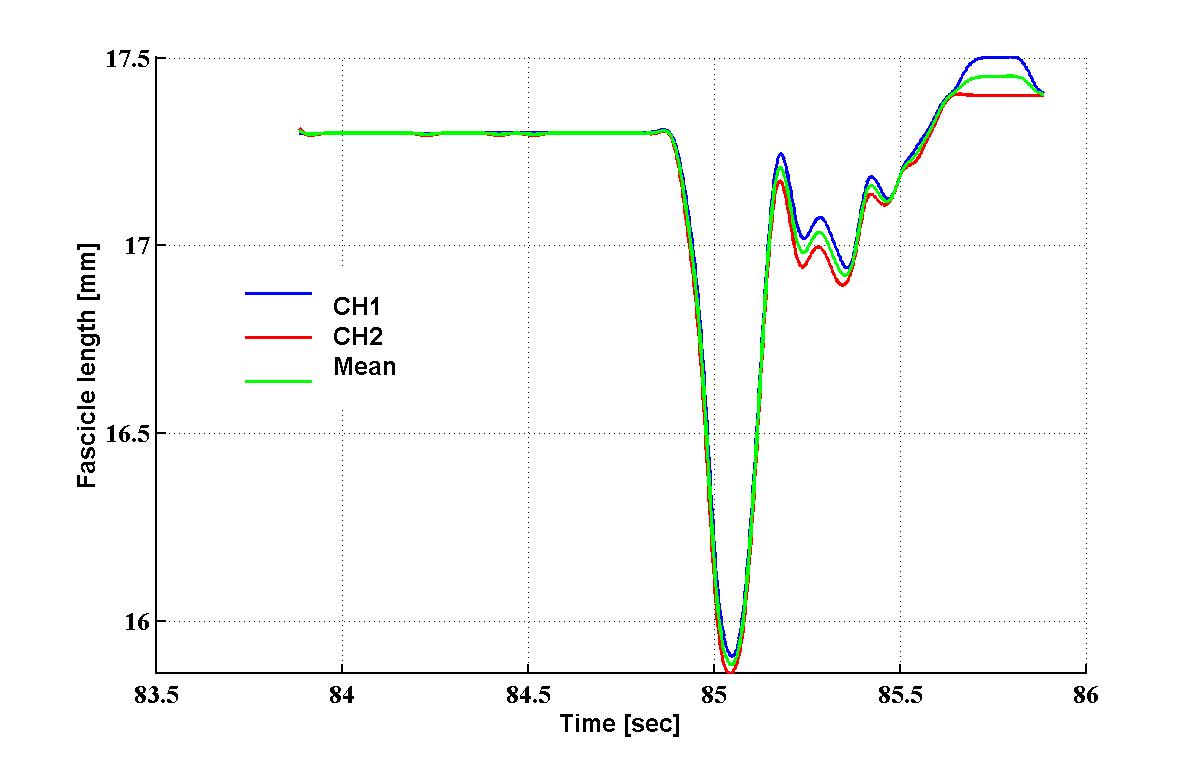

Supplement: S3 File — Full raw data set of the current study. (ZIP) [file pone.0173415.s003.zip › Frog_jumping_full_dataset2/Extracted_data/Frog2/Jump2/blacksono.jpg]

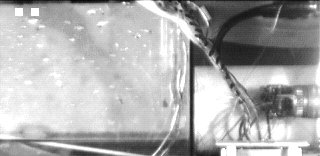

Supplement: S3 File — Full raw data set of the current study. (ZIP) [file pone.0173415.s003.zip › Frog_jumping_full_dataset2/Extracted_data/Frog2/Jump2/end pic.jpg]

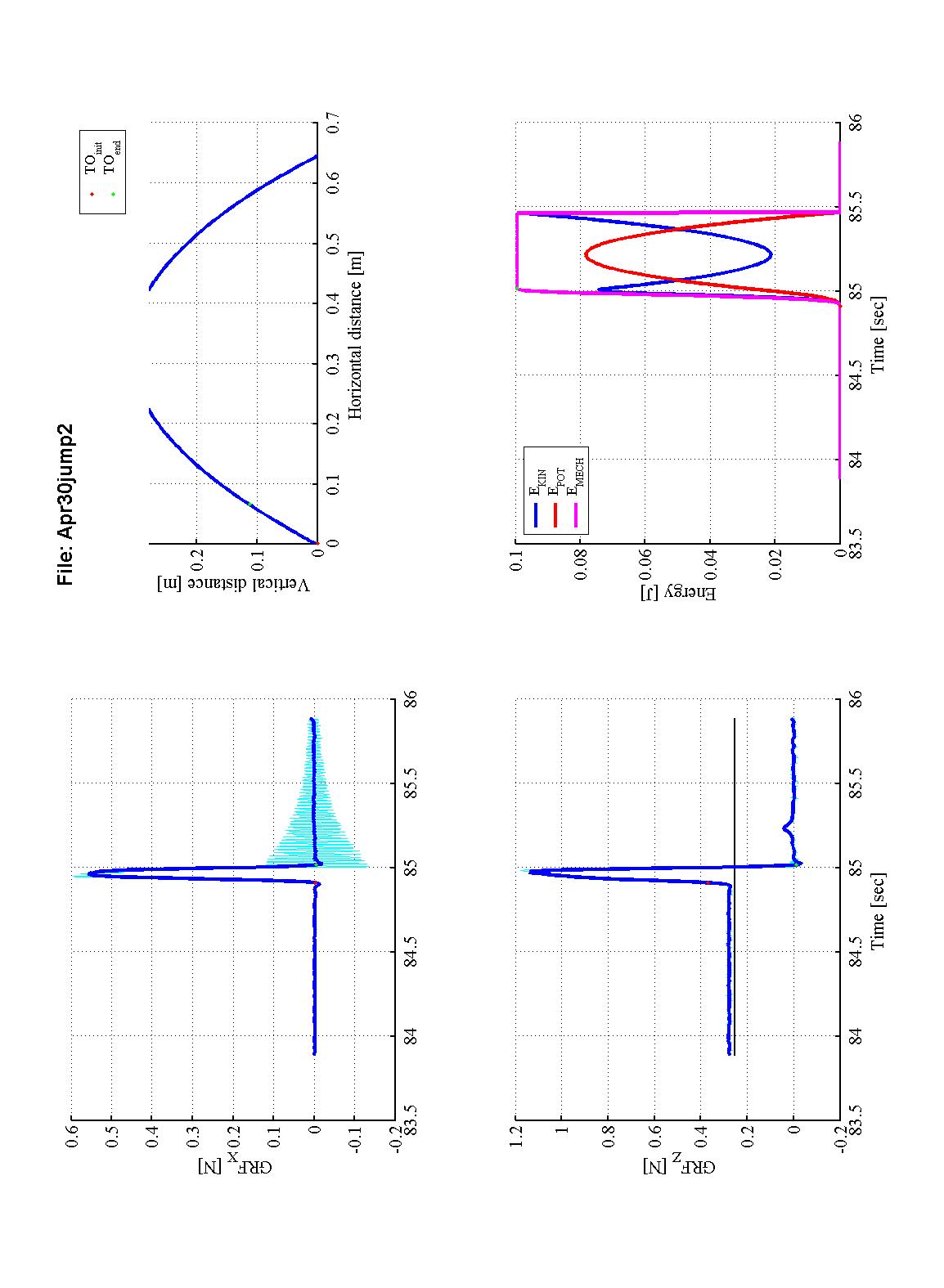

Supplement: S3 File — Full raw data set of the current study. (ZIP) [file pone.0173415.s003.zip › Frog_jumping_full_dataset2/Extracted_data/Frog2/Jump2/fp-dis-en.jpg]

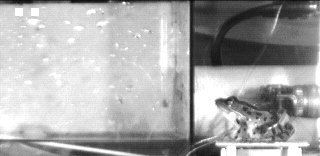

Supplement: S3 File — Full raw data set of the current study. (ZIP) [file pone.0173415.s003.zip › Frog_jumping_full_dataset2/Extracted_data/Frog2/Jump2/initial pic.jpg]

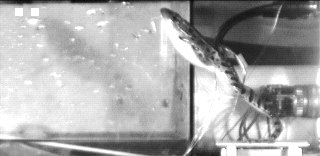

Supplement: S3 File — Full raw data set of the current study. (ZIP) [file pone.0173415.s003.zip › Frog_jumping_full_dataset2/Extracted_data/Frog2/Jump2/peak pic.jpg]

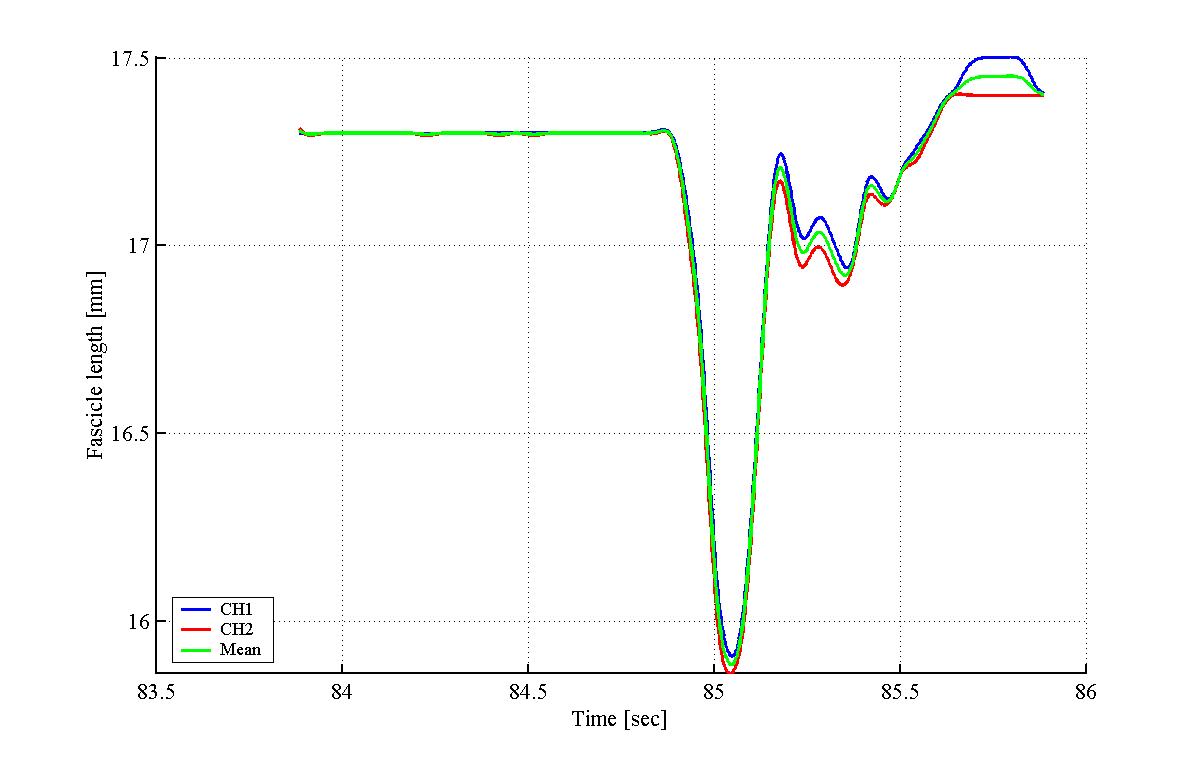

Supplement: S3 File — Full raw data set of the current study. (ZIP) [file pone.0173415.s003.zip › Frog_jumping_full_dataset2/Extracted_data/Frog2/Jump2/sono.jpg]

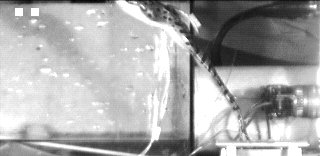

Supplement: S3 File — Full raw data set of the current study. (ZIP) [file pone.0173415.s003.zip › Frog_jumping_full_dataset2/Extracted_data/Frog2/Jump3/end phase.jpg]

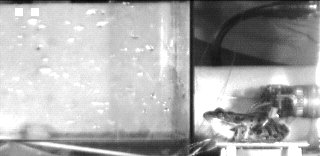

Supplement: S3 File — Full raw data set of the current study. (ZIP) [file pone.0173415.s003.zip › Frog_jumping_full_dataset2/Extracted_data/Frog2/Jump3/initial phase.jpg]

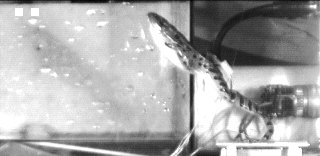

Supplement: S3 File — Full raw data set of the current study. (ZIP) [file pone.0173415.s003.zip › Frog_jumping_full_dataset2/Extracted_data/Frog2/Jump3/peak force.jpg]

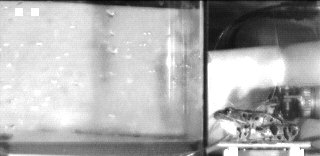

Supplement: S3 File — Full raw data set of the current study. (ZIP) [file pone.0173415.s003.zip › Frog_jumping_full_dataset2/high_speed_video/Frog1/jump2_pics/0ms.jpg]

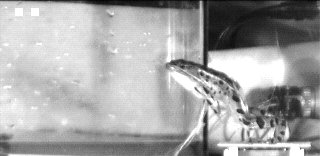

Supplement: S3 File — Full raw data set of the current study. (ZIP) [file pone.0173415.s003.zip › Frog_jumping_full_dataset2/high_speed_video/Frog1/jump2_pics/100ms.jpg]

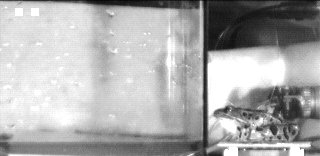

Supplement: S3 File — Full raw data set of the current study. (ZIP) [file pone.0173415.s003.zip › Frog_jumping_full_dataset2/high_speed_video/Frog1/jump2_pics/10ms.jpg]

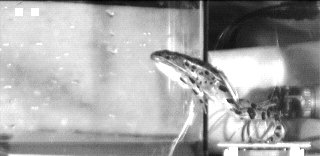

Supplement: S3 File — Full raw data set of the current study. (ZIP) [file pone.0173415.s003.zip › Frog_jumping_full_dataset2/high_speed_video/Frog1/jump2_pics/110ms.jpg]

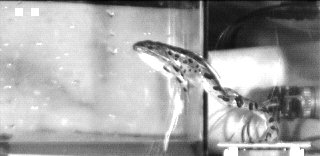

Supplement: S3 File — Full raw data set of the current study. (ZIP) [file pone.0173415.s003.zip › Frog_jumping_full_dataset2/high_speed_video/Frog1/jump2_pics/120ms.jpg]

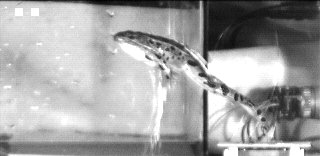

Supplement: S3 File — Full raw data set of the current study. (ZIP) [file pone.0173415.s003.zip › Frog_jumping_full_dataset2/high_speed_video/Frog1/jump2_pics/130ms.jpg]

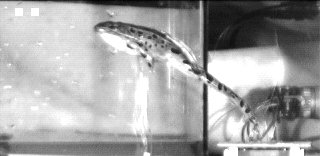

Supplement: S3 File — Full raw data set of the current study. (ZIP) [file pone.0173415.s003.zip › Frog_jumping_full_dataset2/high_speed_video/Frog1/jump2_pics/140ms.jpg]

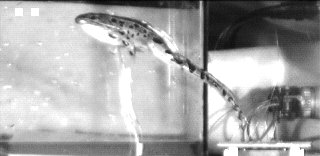

Supplement: S3 File — Full raw data set of the current study. (ZIP) [file pone.0173415.s003.zip › Frog_jumping_full_dataset2/high_speed_video/Frog1/jump2_pics/150ms.jpg]

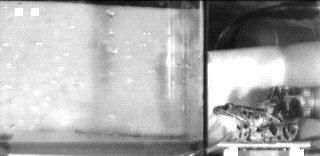

Supplement: S3 File — Full raw data set of the current study. (ZIP) [file pone.0173415.s003.zip › Frog_jumping_full_dataset2/high_speed_video/Frog1/jump2_pics/20ms.jpg]

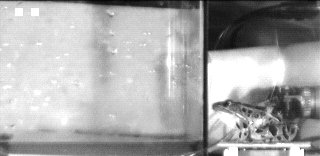

Supplement: S3 File — Full raw data set of the current study. (ZIP) [file pone.0173415.s003.zip › Frog_jumping_full_dataset2/high_speed_video/Frog1/jump2_pics/30ms.jpg]

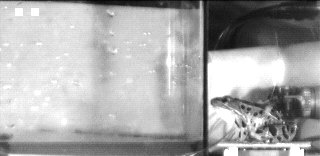

Supplement: S3 File — Full raw data set of the current study. (ZIP) [file pone.0173415.s003.zip › Frog_jumping_full_dataset2/high_speed_video/Frog1/jump2_pics/40ms.jpg]

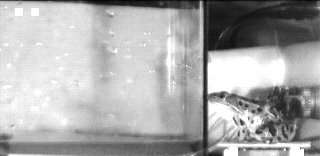

Supplement: S3 File — Full raw data set of the current study. (ZIP) [file pone.0173415.s003.zip › Frog_jumping_full_dataset2/high_speed_video/Frog1/jump2_pics/50ms.jpg]

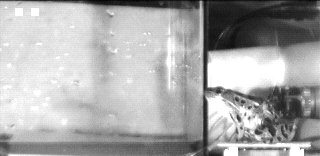

Supplement: S3 File — Full raw data set of the current study. (ZIP) [file pone.0173415.s003.zip › Frog_jumping_full_dataset2/high_speed_video/Frog1/jump2_pics/60ms.jpg]

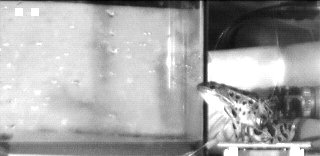

Supplement: S3 File — Full raw data set of the current study. (ZIP) [file pone.0173415.s003.zip › Frog_jumping_full_dataset2/high_speed_video/Frog1/jump2_pics/70ms.jpg]

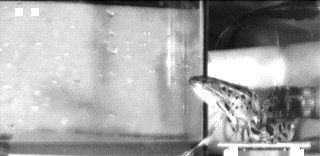

Supplement: S3 File — Full raw data set of the current study. (ZIP) [file pone.0173415.s003.zip › Frog_jumping_full_dataset2/high_speed_video/Frog1/jump2_pics/80ms.jpg]

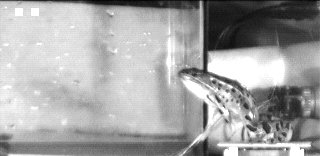

Supplement: S3 File — Full raw data set of the current study. (ZIP) [file pone.0173415.s003.zip › Frog_jumping_full_dataset2/high_speed_video/Frog1/jump2_pics/90ms.jpg]

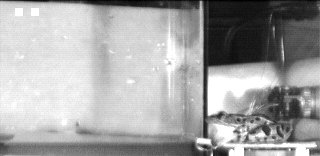

Supplement: S3 File — Full raw data set of the current study. (ZIP) [file pone.0173415.s003.zip › Frog_jumping_full_dataset2/high_speed_video/Frog1/jump6_pics/0ms.jpg]

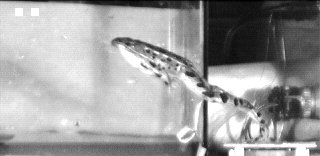

Supplement: S3 File — Full raw data set of the current study. (ZIP) [file pone.0173415.s003.zip › Frog_jumping_full_dataset2/high_speed_video/Frog1/jump6_pics/100ms.jpg]

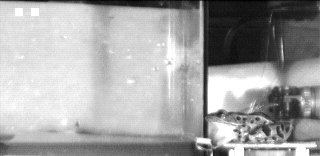

Supplement: S3 File — Full raw data set of the current study. (ZIP) [file pone.0173415.s003.zip › Frog_jumping_full_dataset2/high_speed_video/Frog1/jump6_pics/10ms.jpg]

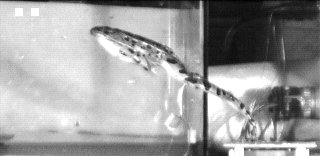

Supplement: S3 File — Full raw data set of the current study. (ZIP) [file pone.0173415.s003.zip › Frog_jumping_full_dataset2/high_speed_video/Frog1/jump6_pics/110ms.jpg]

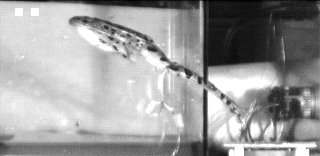

Supplement: S3 File — Full raw data set of the current study. (ZIP) [file pone.0173415.s003.zip › Frog_jumping_full_dataset2/high_speed_video/Frog1/jump6_pics/120ms.jpg]

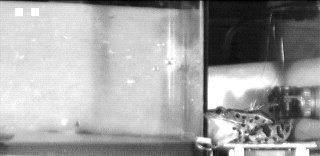

Supplement: S3 File — Full raw data set of the current study. (ZIP) [file pone.0173415.s003.zip › Frog_jumping_full_dataset2/high_speed_video/Frog1/jump6_pics/20ms.jpg]

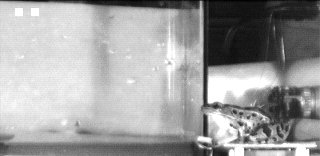

Supplement: S3 File — Full raw data set of the current study. (ZIP) [file pone.0173415.s003.zip › Frog_jumping_full_dataset2/high_speed_video/Frog1/jump6_pics/30ms.jpg]

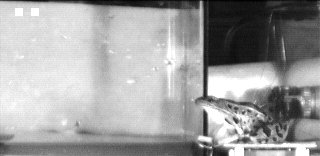

Supplement: S3 File — Full raw data set of the current study. (ZIP) [file pone.0173415.s003.zip › Frog_jumping_full_dataset2/high_speed_video/Frog1/jump6_pics/40ms.jpg]

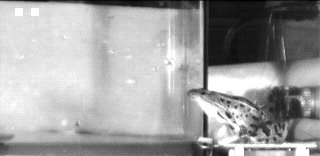

Supplement: S3 File — Full raw data set of the current study. (ZIP) [file pone.0173415.s003.zip › Frog_jumping_full_dataset2/high_speed_video/Frog1/jump6_pics/50ms.jpg]

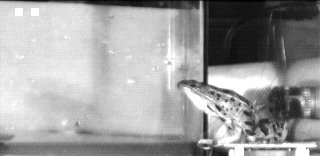

Supplement: S3 File — Full raw data set of the current study. (ZIP) [file pone.0173415.s003.zip › Frog_jumping_full_dataset2/high_speed_video/Frog1/jump6_pics/60ms.jpg]

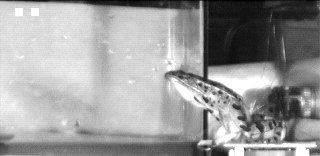

Supplement: S3 File — Full raw data set of the current study. (ZIP) [file pone.0173415.s003.zip › Frog_jumping_full_dataset2/high_speed_video/Frog1/jump6_pics/70ms.jpg]

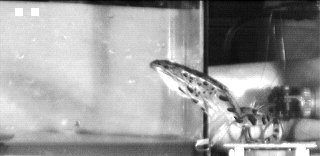

Supplement: S3 File — Full raw data set of the current study. (ZIP) [file pone.0173415.s003.zip › Frog_jumping_full_dataset2/high_speed_video/Frog1/jump6_pics/80ms.jpg]

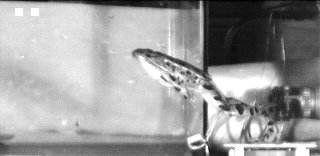

Supplement: S3 File — Full raw data set of the current study. (ZIP) [file pone.0173415.s003.zip › Frog_jumping_full_dataset2/high_speed_video/Frog1/jump6_pics/90ms.jpg]

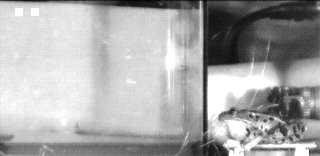

Supplement: S3 File — Full raw data set of the current study. (ZIP) [file pone.0173415.s003.zip › Frog_jumping_full_dataset2/high_speed_video/Frog1/jump8_pics/0ms.jpg]

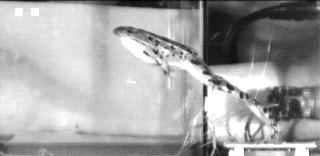

Supplement: S3 File — Full raw data set of the current study. (ZIP) [file pone.0173415.s003.zip › Frog_jumping_full_dataset2/high_speed_video/Frog1/jump8_pics/100ms.jpg]

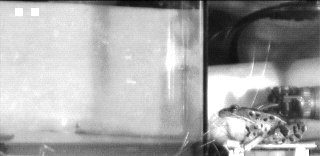

Supplement: S3 File — Full raw data set of the current study. (ZIP) [file pone.0173415.s003.zip › Frog_jumping_full_dataset2/high_speed_video/Frog1/jump8_pics/10ms.jpg]

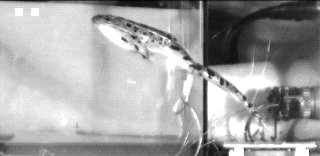

Supplement: S3 File — Full raw data set of the current study. (ZIP) [file pone.0173415.s003.zip › Frog_jumping_full_dataset2/high_speed_video/Frog1/jump8_pics/110ms.jpg]

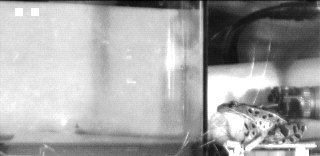

Supplement: S3 File — Full raw data set of the current study. (ZIP) [file pone.0173415.s003.zip › Frog_jumping_full_dataset2/high_speed_video/Frog1/jump8_pics/20ms.jpg]

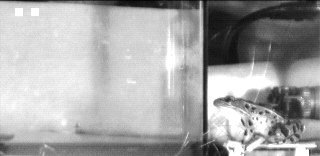

Supplement: S3 File — Full raw data set of the current study. (ZIP) [file pone.0173415.s003.zip › Frog_jumping_full_dataset2/high_speed_video/Frog1/jump8_pics/30ms.jpg]

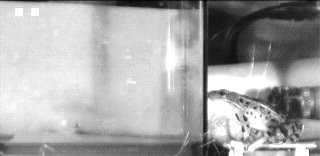

Supplement: S3 File — Full raw data set of the current study. (ZIP) [file pone.0173415.s003.zip › Frog_jumping_full_dataset2/high_speed_video/Frog1/jump8_pics/40ms.jpg]

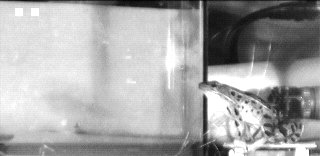

Supplement: S3 File — Full raw data set of the current study. (ZIP) [file pone.0173415.s003.zip › Frog_jumping_full_dataset2/high_speed_video/Frog1/jump8_pics/50ms.jpg]

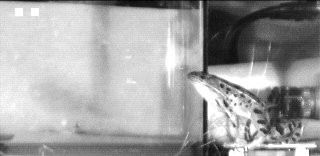

Supplement: S3 File — Full raw data set of the current study. (ZIP) [file pone.0173415.s003.zip › Frog_jumping_full_dataset2/high_speed_video/Frog1/jump8_pics/60ms.jpg]

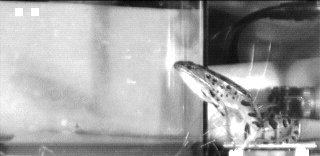

Supplement: S3 File — Full raw data set of the current study. (ZIP) [file pone.0173415.s003.zip › Frog_jumping_full_dataset2/high_speed_video/Frog1/jump8_pics/70ms.jpg]

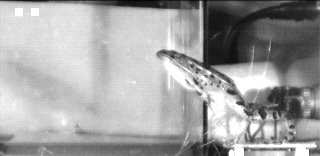

Supplement: S3 File — Full raw data set of the current study. (ZIP) [file pone.0173415.s003.zip › Frog_jumping_full_dataset2/high_speed_video/Frog1/jump8_pics/80ms.jpg]

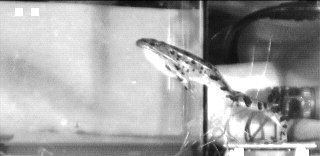

Supplement: S3 File — Full raw data set of the current study. (ZIP) [file pone.0173415.s003.zip › Frog_jumping_full_dataset2/high_speed_video/Frog1/jump8_pics/90ms.jpg]

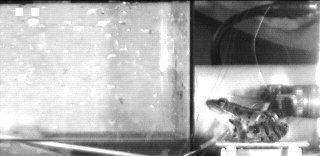

Supplement: S3 File — Full raw data set of the current study. (ZIP) [file pone.0173415.s003.zip › Frog_jumping_full_dataset2/high_speed_video/Frog2/jump1_pics/0ms.jpg]

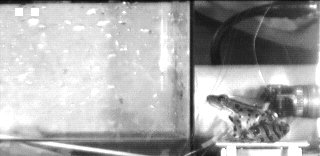

Supplement: S3 File — Full raw data set of the current study. (ZIP) [file pone.0173415.s003.zip › Frog_jumping_full_dataset2/high_speed_video/Frog2/jump1_pics/10ms.jpg]

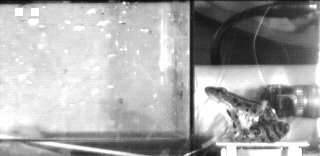

Supplement: S3 File — Full raw data set of the current study. (ZIP) [file pone.0173415.s003.zip › Frog_jumping_full_dataset2/high_speed_video/Frog2/jump1_pics/20ms.jpg]

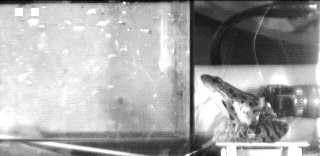

Supplement: S3 File — Full raw data set of the current study. (ZIP) [file pone.0173415.s003.zip › Frog_jumping_full_dataset2/high_speed_video/Frog2/jump1_pics/30ms.jpg]

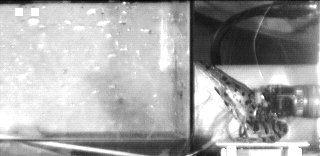

Supplement: S3 File — Full raw data set of the current study. (ZIP) [file pone.0173415.s003.zip › Frog_jumping_full_dataset2/high_speed_video/Frog2/jump1_pics/40ms.jpg]

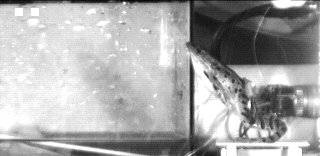

Supplement: S3 File — Full raw data set of the current study. (ZIP) [file pone.0173415.s003.zip › Frog_jumping_full_dataset2/high_speed_video/Frog2/jump1_pics/50ms.jpg]

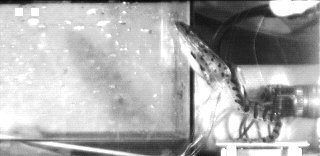

Supplement: S3 File — Full raw data set of the current study. (ZIP) [file pone.0173415.s003.zip › Frog_jumping_full_dataset2/high_speed_video/Frog2/jump1_pics/60ms.jpg]

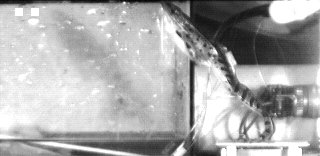

Supplement: S3 File — Full raw data set of the current study. (ZIP) [file pone.0173415.s003.zip › Frog_jumping_full_dataset2/high_speed_video/Frog2/jump1_pics/70ms.jpg]

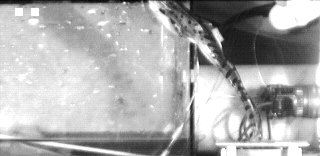

Supplement: S3 File — Full raw data set of the current study. (ZIP) [file pone.0173415.s003.zip › Frog_jumping_full_dataset2/high_speed_video/Frog2/jump1_pics/80ms.jpg]

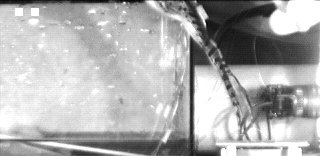

Supplement: S3 File — Full raw data set of the current study. (ZIP) [file pone.0173415.s003.zip › Frog_jumping_full_dataset2/high_speed_video/Frog2/jump1_pics/90ms.jpg]

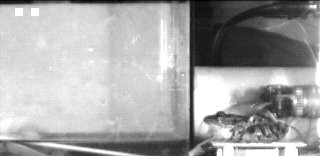

Supplement: S3 File — Full raw data set of the current study. (ZIP) [file pone.0173415.s003.zip › Frog_jumping_full_dataset2/high_speed_video/Frog2/jump11_pics/0ms.jpg]

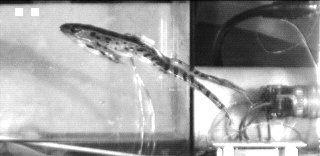

Supplement: S3 File — Full raw data set of the current study. (ZIP) [file pone.0173415.s003.zip › Frog_jumping_full_dataset2/high_speed_video/Frog2/jump11_pics/100ms.jpg]

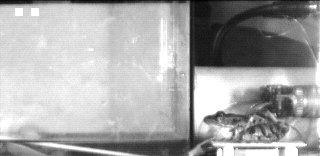

Supplement: S3 File — Full raw data set of the current study. (ZIP) [file pone.0173415.s003.zip › Frog_jumping_full_dataset2/high_speed_video/Frog2/jump11_pics/10ms.jpg]

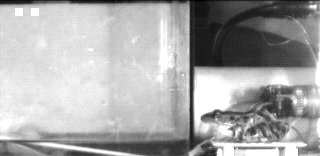

Supplement: S3 File — Full raw data set of the current study. (ZIP) [file pone.0173415.s003.zip › Frog_jumping_full_dataset2/high_speed_video/Frog2/jump11_pics/20ms.jpg]

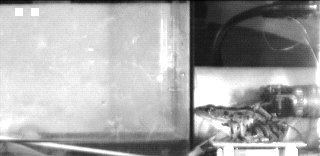

Supplement: S3 File — Full raw data set of the current study. (ZIP) [file pone.0173415.s003.zip › Frog_jumping_full_dataset2/high_speed_video/Frog2/jump11_pics/30ms.jpg]

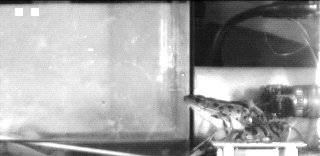

Supplement: S3 File — Full raw data set of the current study. (ZIP) [file pone.0173415.s003.zip › Frog_jumping_full_dataset2/high_speed_video/Frog2/jump11_pics/40ms.jpg]

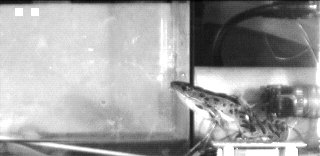

Supplement: S3 File — Full raw data set of the current study. (ZIP) [file pone.0173415.s003.zip › Frog_jumping_full_dataset2/high_speed_video/Frog2/jump11_pics/50ms.jpg]

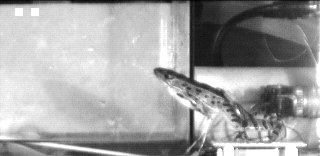

Supplement: S3 File — Full raw data set of the current study. (ZIP) [file pone.0173415.s003.zip › Frog_jumping_full_dataset2/high_speed_video/Frog2/jump11_pics/60ms.jpg]

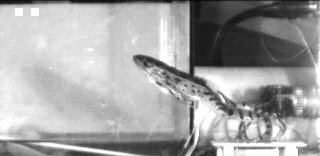

Supplement: S3 File — Full raw data set of the current study. (ZIP) [file pone.0173415.s003.zip › Frog_jumping_full_dataset2/high_speed_video/Frog2/jump11_pics/70ms.jpg]

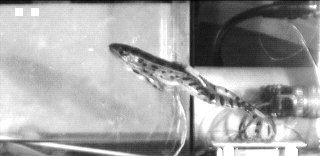

Supplement: S3 File — Full raw data set of the current study. (ZIP) [file pone.0173415.s003.zip › Frog_jumping_full_dataset2/high_speed_video/Frog2/jump11_pics/80ms.jpg]

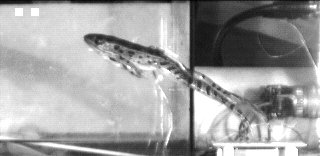

Supplement: S3 File — Full raw data set of the current study. (ZIP) [file pone.0173415.s003.zip › Frog_jumping_full_dataset2/high_speed_video/Frog2/jump11_pics/90ms.jpg]

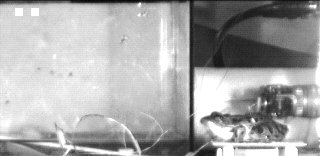

Supplement: S3 File — Full raw data set of the current study. (ZIP) [file pone.0173415.s003.zip › Frog_jumping_full_dataset2/high_speed_video/Frog2/jump12_pics/0ms.jpg]

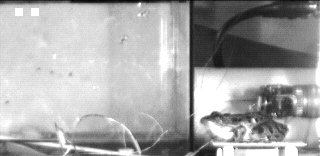

Supplement: S3 File — Full raw data set of the current study. (ZIP) [file pone.0173415.s003.zip › Frog_jumping_full_dataset2/high_speed_video/Frog2/jump12_pics/10ms.jpg]

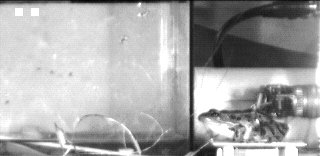

Supplement: S3 File — Full raw data set of the current study. (ZIP) [file pone.0173415.s003.zip › Frog_jumping_full_dataset2/high_speed_video/Frog2/jump12_pics/20ms.jpg]

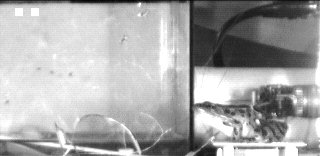

Supplement: S3 File — Full raw data set of the current study. (ZIP) [file pone.0173415.s003.zip › Frog_jumping_full_dataset2/high_speed_video/Frog2/jump12_pics/30ms.jpg]

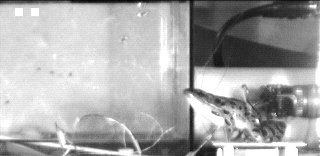

Supplement: S3 File — Full raw data set of the current study. (ZIP) [file pone.0173415.s003.zip › Frog_jumping_full_dataset2/high_speed_video/Frog2/jump12_pics/40ms.jpg]

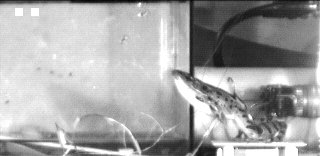

Supplement: S3 File — Full raw data set of the current study. (ZIP) [file pone.0173415.s003.zip › Frog_jumping_full_dataset2/high_speed_video/Frog2/jump12_pics/50ms.jpg]

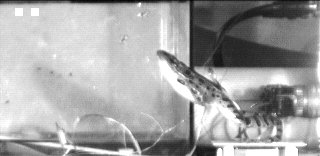

Supplement: S3 File — Full raw data set of the current study. (ZIP) [file pone.0173415.s003.zip › Frog_jumping_full_dataset2/high_speed_video/Frog2/jump12_pics/60ms.jpg]

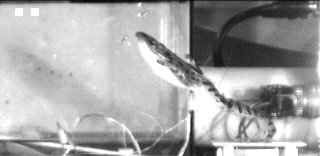

Supplement: S3 File — Full raw data set of the current study. (ZIP) [file pone.0173415.s003.zip › Frog_jumping_full_dataset2/high_speed_video/Frog2/jump12_pics/70ms.jpg]

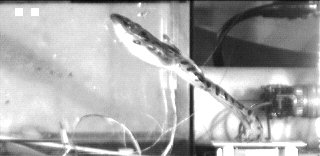

Supplement: S3 File — Full raw data set of the current study. (ZIP) [file pone.0173415.s003.zip › Frog_jumping_full_dataset2/high_speed_video/Frog2/jump12_pics/80ms.jpg]

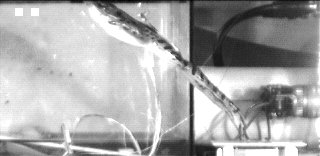

Supplement: S3 File — Full raw data set of the current study. (ZIP) [file pone.0173415.s003.zip › Frog_jumping_full_dataset2/high_speed_video/Frog2/jump12_pics/90ms.jpg]

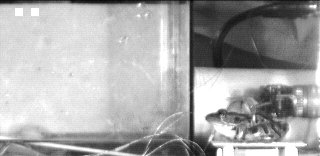

Supplement: S3 File — Full raw data set of the current study. (ZIP) [file pone.0173415.s003.zip › Frog_jumping_full_dataset2/high_speed_video/Frog2/jump13_pics/0ms.jpg]

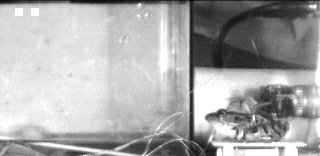

Supplement: S3 File — Full raw data set of the current study. (ZIP) [file pone.0173415.s003.zip › Frog_jumping_full_dataset2/high_speed_video/Frog2/jump13_pics/10ms.jpg]

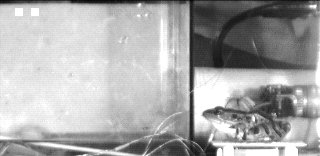

Supplement: S3 File — Full raw data set of the current study. (ZIP) [file pone.0173415.s003.zip › Frog_jumping_full_dataset2/high_speed_video/Frog2/jump13_pics/20ms.jpg]

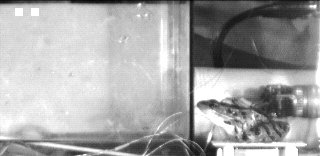

Supplement: S3 File — Full raw data set of the current study. (ZIP) [file pone.0173415.s003.zip › Frog_jumping_full_dataset2/high_speed_video/Frog2/jump13_pics/30ms.jpg]

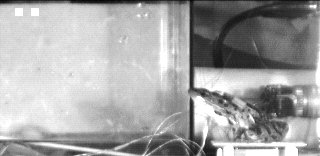

Supplement: S3 File — Full raw data set of the current study. (ZIP) [file pone.0173415.s003.zip › Frog_jumping_full_dataset2/high_speed_video/Frog2/jump13_pics/40ms.jpg]
