# Supplementary material for: In vivo muscle force and muscle power during near-maximal frog jumps
Source: PLoS One. 2017 Mar 10;12(3):e0173415. doi: 10.1371/journal.pone.0173415 (PMC5345813; doi:10.1371/journal.pone.0173415)
Supplement: S3 File — Full raw data set of the current study. (ZIP) [file pone.0173415.s003.zip › Frog_jumping_full_dataset2/Extracted_data/Frog2/Jump2/Jump sequence pics.doc]

**Initial 0 ms	Peak Force 74 ms	End 104 ms**
